# Non-Professional Criticism in Medical Matters

**Published:** 1882-11

**Authors:** 


### NON-PROFESSIONAL CRITICISM IN MEDICAL MATTERS.

We notice in a recent issue of a monthly paper published in this city. which professes devotion to mercantile interests, and which essays the discussion of commercial problems, a very harsh and unjust criticism of the views expressed by a correspondent in the last number of The Register concerning the relations which exist between specialists, general practitioners and the code of medical ethics.

Of course the writer of this article is entitled to his opinion, and his opinion on this subject is entitled to about as much respect as the expression of his views on any other subject of which he knows as little. Hence, the declaration of the sentiments therein contained is here referred to only as fairly representing the ignorance and prejudice which obtain among many intelligent persons regarding what is commonly termed, sometimes with disdainful intonation, medical etiquette, and which differs in no essential respect from the deportment recognized as incumbent upon gentlemen in any of the relations of life.

Medical ethics, like other just laws, possesses no terrors for the upright. The observance of its provisions is purely voluntary, and its reasonable requirements are strictly in accord with the instinctive feelings of every conscientions and honorable man.

The "code" is only obnoxious to those who seek to retain respectable association, and, while reaping the benefits of that association, are guilty of the ordinary and disreputable practices of empirics.

The effort in the article before us to make it appear that the communication in question bore special reference to any particular individual is the very essence of child-like innocence.

